# Supplementary material for: Discovery and application of insertion-deletion (INDEL) polymorphisms for QTL mapping of early life-history traits in Atlantic salmon
Source: BMC Genomics. 2010 Mar 8;11:156. doi: 10.1186/1471-2164-11-156 (PMC2838853; doi:10.1186/1471-2164-11-156)
Supplement: Additional file 2 — Information on developed 76 locus single-run INDEL panel in Atlantic salmon. Information on fluorescence labeling, primer concentrations, PCR pooling and links to alignments, INDEL motifs and GENESCAN (Burge and Karlin 1997) predictions of genes/exons are available in html format. [file 1471-2164-11-156-S2.ZIP › Additionalfile2/snpsummary974.html]

```
Cluster 120 Contig 2

prev  Summary    Contig List  next
```

Size of Consensus sequence = 1350

Number of sequences = 55

Minimum redundancy = 6

Key

A gi|117441969|gb|EG774192.1|EG774192 EST\_ssal\_evd\_49557 ssalevd thymus Salmo salar cDNA Salmo salar cDNA clone ssal\_evd\_566\_259\_rev 5', mRNA sequence  
B gi|117441970|gb|EG774193.1|EG774193 EST\_ssal\_evd\_49558 ssalevd thymus Salmo salar cDNA Salmo salar cDNA clone ssal\_evd\_566\_259\_fwd 3', mRNA sequence  
C gi|24337341|gb|CA037846.1|CA037846 ssallna007053 liver Salmo salar cDNA, mRNA sequence  
D gi|117444420|gb|EG776643.1|EG776643 EST\_ssal\_evd\_51764 ssalevd thymus Salmo salar cDNA Salmo salar cDNA clone ssal\_evd\_569\_288\_rev 5', mRNA sequence  
E gi|117444419|gb|EG776642.1|EG776642 EST\_ssal\_evd\_51763 ssalevd thymus Salmo salar cDNA Salmo salar cDNA clone ssal\_evd\_569\_288\_fwd 3', mRNA sequence  
F gi|29311728|gb|CB500502.1|CB500502 ssalga506085 head Salmo salar cDNA, mRNA sequence  
G gi|117547285|gb|EG878730.1|EG878730 EST\_ssal\_eve\_22218 ssaleve thyroid Salmo salar cDNA Salmo salar cDNA clone ssal\_eve\_530\_086\_rev 5', mRNA sequence  
H gi|45307570|gb|CK877939.1|CK877939 SGP139285 Atlantic salmon Gills cDNA library Salmo salar cDNA clone G6-0707 5', mRNA sequence  
I gi|24353422|gb|CA047252.1|CA047252 ssalga006077 head Salmo salar cDNA, mRNA sequence  
J gi|117479873|gb|EG812090.1|EG812090 EST\_ssal\_evd\_32201 ssalevd thymus Salmo salar cDNA Salmo salar cDNA clone ssal\_evd\_542\_280\_rev 5', mRNA sequence  
K gi|25997273|gb|CA768018.1|CA768018 ssalplnb504023 gut Salmo salar cDNA, mRNA sequence  
L gi|29312844|gb|CB501618.1|CB501618 ssalga508081 head Salmo salar cDNA, mRNA sequence  
M gi|84983477|gb|DW533827.1|DW533827 EST\_ssal\_plnb\_2463 plnb Salmo salar cDNA clone ssal\_plnb\_019\_007\_fwd 3', mRNA sequence  
N gi|117824840|gb|EG897536.1|EG897536 EST\_ssal\_evf\_36683 ssalevf mixed\_tissue Salmo salar cDNA Salmo salar cDNA clone ssal\_evf\_548\_332\_fwd 3', mRNA sequence  
O gi|117857462|gb|EG930158.1|EG930158 EST\_ssal\_evf\_32300 ssalevf mixed\_tissue Salmo salar cDNA Salmo salar cDNA clone ssal\_evf\_542\_354\_rev 5', mRNA sequence  
P gi|117476629|gb|EG808848.1|EG808848 EST\_ssal\_evd\_29283 ssalevd thymus Salmo salar cDNA Salmo salar cDNA clone ssal\_evd\_538\_149\_rev 5', mRNA sequence  
Q gi|24352589|gb|CA046419.1|CA046419 ssalbrh007085 head Salmo salar cDNA, mRNA sequence  
R gi|117861340|gb|EG934036.1|EG934036 EST\_ssal\_evf\_35790 ssalevf mixed\_tissue Salmo salar cDNA Salmo salar cDNA clone ssal\_evf\_547\_250\_rev 5', mRNA sequence  
S gi|117870222|gb|EG942918.1|EG942918 EST\_ssal\_evf\_26195 ssalevf mixed\_tissue Salmo salar cDNA Salmo salar cDNA clone ssal\_evf\_534\_263\_rev 5', mRNA sequence  
T gi|117423604|gb|EG755828.1|EG755828 EST\_ssal\_plnb\_6927 ssalplnb mixed\_tissue Salmo salar cDNA Salmo salar cDNA clone ssal\_plnb\_029\_354\_fwd 3', mRNA sequence  
U gi|29321276|gb|CB510050.1|CB510050 ssalnwh501208 whole Salmo salar cDNA, mRNA sequence  
V gi|117454226|gb|EG786445.1|EG786445 EST\_ssal\_evd\_53215 ssalevd thymus Salmo salar cDNA Salmo salar cDNA clone ssal\_evd\_571\_277\_rev 5', mRNA sequence  
W gi|117499101|gb|EG831318.1|EG831318 EST\_ssal\_eve\_43479 ssaleve thyroid Salmo salar cDNA Salmo salar cDNA clone ssal\_eve\_559\_044\_rev 5', mRNA sequence  
X gi|117523011|gb|EG854738.1|EG854738 EST\_ssal\_eve\_15457 ssaleve thyroid Salmo salar cDNA Salmo salar cDNA clone ssal\_eve\_521\_001\_rev 5', mRNA sequence  
Y gi|117836748|gb|EG909444.1|EG909444 EST\_ssal\_evf\_11657 ssalevf mixed\_tissue Salmo salar cDNA Salmo salar cDNA clone ssal\_evf\_514\_015\_rev 5', mRNA sequence  
Z gi|29313325|gb|CB502099.1|CB502099 ssalmge501009 gut Salmo salar cDNA, mRNA sequence  
a gi|117563656|gb|EG884632.1|EG884632 EST\_ssal\_evf\_43069 ssalevf mixed\_tissue Salmo salar cDNA Salmo salar cDNA clone ssal\_evf\_557\_215\_rev 5', mRNA sequence  
b gi|117522027|gb|EG853754.1|EG853754 EST\_ssal\_eve\_14571 ssaleve thyroid Salmo salar cDNA Salmo salar cDNA clone ssal\_eve\_519\_303\_rev 5', mRNA sequence  
c gi|24356052|gb|CA049882.1|CA049882 ssalsrkh002039 kidney Salmo salar cDNA, mRNA sequence  
d gi|24356125|gb|CA049955.1|CA049955 ssalsrkh002082 kidney Salmo salar cDNA, mRNA sequence  
e gi|117442078|gb|EG774301.1|EG774301 EST\_ssal\_evd\_49655 ssalevd thymus Salmo salar cDNA Salmo salar cDNA clone ssal\_evd\_566\_311\_fwd 3', mRNA sequence  
f gi|24354260|gb|CA048090.1|CA048090 ssalsrkb001078 kidney Salmo salar cDNA, mRNA sequence  
g gi|29312110|gb|CB500884.1|CB500884 ssalga501051 head Salmo salar cDNA, mRNA sequence  
h gi|22033620|gb|AJ425452.1|AJ425452 AJ425452 Salmo salar spleen adult Salmo salar cDNA clone s14A08, mRNA sequence  
i gi|89830275|gb|DY692647.1|DY692647 EST\_ssal\_plnb\_5557 ssalplnb mixed\_tissue Salmo salar cDNA Salmo salar cDNA clone ssal\_plnb\_027\_062\_fwd 3', mRNA sequence  
j gi|45306157|gb|CK876526.1|CK876526 SGP137807 Atlantic salmon Eye cDNA library Salmo salar cDNA clone OY4-0684 5', mRNA sequence  
k gi|45311131|gb|CK881500.1|CK881500 SGP141842 Atlantic salmon Head kidney cDNA library Salmo salar cDNA clone FN4-0950 5', mRNA sequence  
l gi|117563655|gb|EG884631.1|EG884631 EST\_ssal\_evf\_43068 ssalevf mixed\_tissue Salmo salar cDNA Salmo salar cDNA clone ssal\_evf\_557\_215\_fwd 3', mRNA sequence  
m gi|57126688|gb|CX358129.1|CX358129 ssalrgb519164\_rev\_0 mixed\_tissue Salmo salar cDNA, mRNA sequence  
n gi|117476628|gb|EG808847.1|EG808847 EST\_ssal\_evd\_29282 ssalevd thymus Salmo salar cDNA Salmo salar cDNA clone ssal\_evd\_538\_149\_fwd 3', mRNA sequence  
o gi|117861339|gb|EG934035.1|EG934035 EST\_ssal\_evf\_35789 ssalevf mixed\_tissue Salmo salar cDNA Salmo salar cDNA clone ssal\_evf\_547\_250\_fwd 3', mRNA sequence  
p gi|117870223|gb|EG942919.1|EG942919 EST\_ssal\_evf\_26196 ssalevf mixed\_tissue Salmo salar cDNA Salmo salar cDNA clone ssal\_evf\_534\_263\_fwd 3', mRNA sequence  
q gi|29317172|gb|CB505946.1|CB505946 ssalplnb504340\_rev gut Salmo salar cDNA, mRNA sequence  
r gi|45316407|gb|CK886776.1|CK886776 SGP148661 Atlantic salmon Kidney cDNA library Salmo salar cDNA clone BN7-0823 5', mRNA sequence  
s gi|117423605|gb|EG755829.1|EG755829 EST\_ssal\_plnb\_6928 ssalplnb mixed\_tissue Salmo salar cDNA Salmo salar cDNA clone ssal\_plnb\_029\_354\_rev 5', mRNA sequence  
t gi|89830273|gb|DY692646.1|DY692646 EST\_ssal\_plnb\_5556 ssalplnb mixed\_tissue Salmo salar cDNA Salmo salar cDNA clone ssal\_plnb\_027\_062\_rev 5', mRNA sequence  
u gi|117454237|gb|EG786456.1|EG786456 EST\_ssal\_evd\_53216 ssalevd thymus Salmo salar cDNA Salmo salar cDNA clone ssal\_evd\_571\_277\_fwd 3', mRNA sequence  
v gi|117836747|gb|EG909443.1|EG909443 EST\_ssal\_evf\_11656 ssalevf mixed\_tissue Salmo salar cDNA Salmo salar cDNA clone ssal\_evf\_514\_015\_fwd 3', mRNA sequence  
w gi|84974218|gb|DW472619.1|DW472619 SGP301265 Atlantic salmon Spleen cDNA library Salmo salar cDNA clone MI5-2662 5', mRNA sequence  
x gi|117523012|gb|EG854739.1|EG854739 EST\_ssal\_eve\_15458 ssaleve thyroid Salmo salar cDNA Salmo salar cDNA clone ssal\_eve\_521\_001\_fwd 3', mRNA sequence  
y gi|117479872|gb|EG812089.1|EG812089 EST\_ssal\_evd\_32200 ssalevd thymus Salmo salar cDNA Salmo salar cDNA clone ssal\_evd\_542\_280\_fwd 3', mRNA sequence  
z gi|117547286|gb|EG878731.1|EG878731 EST\_ssal\_eve\_22219 ssaleve thyroid Salmo salar cDNA Salmo salar cDNA clone ssal\_eve\_530\_086\_fwd 3', mRNA sequence  
A gi|117522028|gb|EG853755.1|EG853755 EST\_ssal\_eve\_14572 ssaleve thyroid Salmo salar cDNA Salmo salar cDNA clone ssal\_eve\_519\_303\_fwd 3', mRNA sequence  
B gi|84983476|gb|DW533826.1|DW533826 EST\_ssal\_plnb\_2462 plnb Salmo salar cDNA clone ssal\_plnb\_019\_007\_rev 5', mRNA sequence  
C gi|117499102|gb|EG831319.1|EG831319 EST\_ssal\_eve\_43480 ssaleve thyroid Salmo salar cDNA Salmo salar cDNA clone ssal\_eve\_559\_044\_fwd 3', mRNA sequence

5 SNPs detected

A B C D E F G H I J K L M N O P Q R S T U V W X Y Z a b c d e f g h i j k l m n o p q r s t u v w x y z A B C  cosegregation weighted

651 . . . . . . . . . . . . . - - - T T T T T - T T T T - T T T T T T T T T T - . - T T . . . T - T . T T . T . T   1/5 12.73
652 . . . . . . . . . . . . . - - - T T T T T - T - T T - - - - T T T T T - T - . - T T . . . T - T . - T . - . T   1/5 12.73
689 . . . . . . . . . . . . . A . A . . - A A A - A A A - - A A A A A A - - A - . A - - . . . - A A . A A . - . -   3/5 34.91
690 . . . . . . . . . . . . . A . A . . - A A A - A A A - - A A A A A A - - A - . A - - . . . - A A . A A . - . -   3/5 34.91
691 . . . . . . . . . . . . . A . A . . - A A A - A A A - - A A A A A A - - A - . A - - . . . - A A . A A . - . -   3/5 34.91
